# P-2334. Clesrovimab Binding Site Conservation on the RSV F Protein: An Evaluation of RSV Molecular Sequencing Data from 2019-2023 and GenBank Sequence Analysis

**DOI:** 10.1093/ofid/ofae631.2486

**Published:** 2025-01-29

**Authors:** Arthur Fridman, Latisha Love-Gregory, Radha Railkar, Diane Levitan, Julie Strizki, Kalpit A Vora

**Affiliations:** Merck & Co., Inc., Rahway, New Jersey; Merck & Co., Inc., Rahway, New Jersey; Merck & Co., Inc., Rahway, New Jersey; Merck & Co., Inc., Rahway, New Jersey; Merck & Co., Inc., Rahway, New Jersey; Merck & Co., Inc., Rahway, New Jersey

## Abstract

**Background:**

Clesrovimab is a half-life extended RSV-neutralizing antibody targeting site IV of the RSV fusion protein, that is currently in phase 3 development for the prevention of RSV in infants. As viral sequences drift over time, the possibility for the emergence of viral resistance exists, and the conduct of contemporary global RSV surveillance is important.

**Methods:**

Here, we report the frequency of naturally occurring RSV F gene polymorphisms in the clesrovimab binding site [amino acids (a.a) 426-447] using two approaches. First, a total of 15,527 RSV F protein sequences with a complete extracellular domain were obtained from GenBank (accessed April 2024) for analysis. Sequences were aligned using MUSCLE multiple sequence alignment program. The a.a. frequency at each position was determined as well as the frequency of variants in the clesrovimab binding site. Secondly, we collected RSV positive samples in 2019 through 2023 from individuals of any age in a total of 8 countries (in the northern and southern hemisphere). Next generation sequencing was performed of the RSV F gene and data were analyzed for sequence changes in the clesrovimab binding site.

**Results:**

A total of 15,495 of 15,527 (99.8%) of sequences analyzed from GenBank were 100% conserved at the clesrovimab binding site. Thirteen clesrovimab epitope variants were found, 7 of which occurred in 2 or more sequences. The most frequent variant occurred in < 0.04% of GenBank sequences. Additionally, a total of 555 RSV viruses (300 RSV A [54%], 255 RSV B [46%]) were analyzed from the molecular surveillance. There were no variants identified in the clesrovimab binding site that met the prespecified study criteria (prevalence over 5% AND a variant allele fraction of >10%).

**Conclusion:**

These data demonstrate that the clesrovimab binding epitope on site IV of the RSV fusion protein is highly conserved. These findings are in line with previous reports on the conserved nature of site IV and support the continued development of clesrovimab for the prevention of RSV disease in infants.

**Disclosures:**

Arthur Fridman, PhD, Merck & Co., Inc.: employee|Merck & Co., Inc.: Stocks/Bonds (Public Company) Latisha Love-Gregory, PhD, Merck & Co., Inc.: Employee|Merck & Co., Inc.: Stocks/Bonds (Public Company) Radha Railkar, PhD, Merck & Co., Inc.: Employee|Merck & Co., Inc.: Stocks/Bonds (Public Company) Diane Levitan, PhD, Merck & Co., Inc.: Employee|Merck & Co., Inc.: Stocks/Bonds (Public Company) Julie Strizki, PhD, Merck & Co., Inc.: Employee|Merck & Co., Inc.: Stocks/Bonds (Public Company) Kalpit A. Vora, PhD, Merck & Co., Inc.: Employee|Merck & Co., Inc.: Stocks/Bonds (Public Company)

